# Down Regulation of T Cell Receptor Expression in COPD Pulmonary CD8 Cells

**DOI:** 10.1371/journal.pone.0071629

**Published:** 2013-08-19

**Authors:** Seamus Grundy, Jonathan Plumb, Simon Lea, Manminder Kaur, David Ray, Dave Singh

**Affiliations:** 1 University of Manchester, Manchester Academic Health Science Centre, University Hospital of South Manchester Foundation Trust, Manchester, United Kingdom; 2 School of Medicine and Manchester Academic Health Science Centre, University of Manchester, Manchester, United Kingdom; University of Tübingen, Germany

## Abstract

CD8 cells may contribute towards an autoimmune process in COPD. Down regulation of T cell receptor (TCR) signalling molecules occurs in autoimmune diseases with consequent T cell dysfunction. We hypothesise that TCR signalling is abnormal in COPD pulmonary CD8 cells. Micro-array gene expression analysis of blood and pulmonary COPD CD8 samples was performed and compared to pulmonary CD8 cells from smoker controls (S). We focused on the TCR signalling pathway, with validation of key findings using polymerase chain reaction and immunofluorescence. TCR signalling molecules in COPD pulmonary CD8 cells were down regulated compared to blood CD8 cells (CD247: fold change (FC) −2.43, Q = 0.001; LCK: FC −2.25, Q = 0.01). Micro-array analysis revealed no significant differences between COPD and S pulmonary CD8 cells. However, PCR revealed significantly lower gene expression levels of CD247 (FC −1.79, p = 0.04) and LCK (FC −1.77, p = 0.01) in COPD compared to S pulmonary CD8 cells. CD247 down regulation in COPD CD8 cells was confirmed by immunofluorescent staining of bronchoalveolar lavage cells: Significantly fewer COPD CD8 cells co-expressed CD247 compared to healthy non-smoker CD8 cells (mean 88.9 vs 75.2%, p<0.05) There is down regulation of TCR signalling molecules in COPD pulmonary CD8 cells. This may cause T cell dysfunction.

## Introduction

Chronic obstructive pulmonary disease (COPD) is characterised by poorly reversible airflow obstruction and airway inflammation that develop as a result of an abnormal inflammatory reaction to the inhalation of noxious particles, most commonly cigarette smoke [1]. Not all smokers develop COPD, and the reasons underlying this are poorly understood.

Historically, there has been much focus on the role of the innate immune system in COPD [2], [3]. However, there is increasing evidence of an adaptive immune component to the disease [4], [5]; T cells [6], and in particular CD8 T cells, are increased in number in the lungs of COPD patients [7]–[9]. CD8 cells play an important role in auto-immune diseases such as rheumatoid arthritis, systemic lupus erythematosus and diabetes mellitus [10]. Autoreactive CD8 cells can drive auto-immunity through cytotoxic activity that leads to tissue destruction [11] and consequently increases the exposure of self-antigens. Additionally, CD8 cells produce pro-inflammatory cytokines such as interleukin 2 (IL-2), interferon gamma (IFNγ) and tumour necrosis factor alpha (TNFα) and chemokines such as C-X-C chemokine motif 10 (CXCL10) and chemokine (C-C) motif ligand 5 (CCL5) that recruit other inflammatory cells. COPD CD8 cells release more cytokines [12] and display greater cytotoxic activity compared to controls [13]. It has been proposed that CD8 cells contribute to an auto-immune process in COPD leading to persistent and progressive airway inflammation [14].

The T cell receptor (TCR) is responsible for recognising antigens from pathogens such as viruses, in order to generate an immune response [15]. Auto-immune diseases are associated with down-regulation of constituent molecules of the T cell receptor signalling pathway such as T cell receptor-ζ (CD247) [16] and lymphocyte-specific protein tyrosine kinase (LCK) [17]. This down regulation leads to T cell dysfunction which may have important physiological consequences including an inadequate response to infection [18]. Recurrent airway infection leading to exacerbations occurs in a subset of COPD patients [19]. Altered TCR signalling may be involved in the increased susceptibility to exacerbations that is evident in this COPD phenotype.

In order to further elucidate the role of CD8 cells in COPD, we undertook molecular phenotyping of COPD CD8 cells, using microarray analysis. This revealed decreased expression of key components of the TCR signalling pathway in pulmonary CD8 cells. It was subsequently confirmed using specific polymerase chain reaction (PCR) analysis and protein quantification by immunofluorescence that TCR components including CD247 are down regulated in COPD pulmonary CD8 cells compared to controls.

## Methods

### Subjects

2 separate cohorts of patients were recruited for this study – see table 1 for demography. Lung tissue was obtained from 6 COPD patients and 6 smokers with normal lung function (S) who were undergoing lung resection for confirmed or suspected malignancy; these samples were used for micro-array and PCR analysis. We specifically only included ex-smokers who had stopped smoking for at least one year, in order to avoid a confounding effect of current smoking. Peripheral blood mononuclear cells (PBMCs) were also obtained from these 6 COPD patients for micro-array analysis. Samples that had previously been obtained from 7 COPD patients, 5 S and 8 healthy non-smokers (HNS) who had undergone bronchoscopy to obtain bronchoalveolar lavage (BAL) for research purposes were used for immunoflourescence. All patients gave written informed consent. The study was approved by the local research ethics committee (South Manchester Research Ethics Committee, reference: 03/SM/396).

**Table 1:** Subject demographics.

	Lung tissue samples		BAL samples		
	COPD	S	COPD	S	HNS
**Age**	64.8 (5.0)	73.1 (8.5)	63.1 (53–74)*	43.3 (35–56)	30.7 (24–42)
**M/F**	5/1	4/2	6/1	3/2	5/3
**FEV_1_ (L)**	1.68 (0.59)	2.11 (0.67)	1.81 (0.64)	2.85 (0.81)	3.57 (0.70)
**FEV_1%_ predicted**	60.0 (16.2)*	89.2 (13.9)	59.0 (15.4)*	99.1 (13.2)	107.6 (9.6)
**FEV_1_:FVC**	55.3 (14.4)*	74.2 (2.8)	49.6 (9.2)*	82.8 (3.7)	83.4 (5.1)
**Current smoker**	0	0	7	3	0
**ICS**	1	0	2	0	0
**Smoking history (pk yrs)**	47.3 (18.9)	31.9 (13.9)	54.7 (28.9)	15.7 (10.0)	0

Data are presented as mean (SD) or median (range). BAL: bronchoalveolar lavage; COPD: chronic obstructive pulmonary disease; S: smoker with normal lung function; HNS: healthy non-smoker; M: male; F: female, FEV_1_: forced expiratory volume in 1 second; FVC: forced vital capacity; L: litres; ICS: inhaled corticosteroids; pk yrs: cigarette pack years. Statistically significant differences for COPD compared to S and/or HNS represented by *p<0.05.

### Cell Isolation

PBMCs were obtained from venous blood by Ficoll-paque centrifugation (GE healthcare, Buckinghamshire, UK). CD8 cells were isolated from PBMCs using positive selection CD8 microbeads (Miltenyi Biotec, Surrey, UK).

Pulmonary CD8 cells were isolated from explanted human lung. Briefly, lung tissue was homogenised in a blender (see methods S1 for detailed protocol). Cells were resuspended in isotonic 40% percoll (GE healthcare) and centrifuged over isotonic 70% percoll (GE healthcare) with a population of enriched lymphocytes being obtained at the percoll interface. CD8 cells were isolated from the enriched lymphocytes using positive selection CD8 microbeads (Miltenyi biotec).

BAL was obtained using a flexible bronchoscope whilst volunteers were under conscious sedation. The bronchoscope was wedged sub-segmentally and 240 ml of 0.9% saline was instilled and aspirated from each upper lobe. BAL fluid was filtered (100 µm filter, MIlipore, Watford, UK) and resuspended in RPMI-1640 (Sigma-Aldrich, Dorset, UK) at a concentration of 1×10^6^ total cells/ml. Cytospins were prepared immediately air dried and cells were fixed in frozen methanol (Sigma-Aldrich).

### RNA Extraction

Cells were lysed in RLT buffer (Qiagen, Sussex, UK) +1% β-mercaptoethanol (Sigma-Aldrich) and stored at −80°C. Samples were homogenised with Qiashredder (Qiagen) and ribonucleic acid (RNA) was extracted using RNeasy mini kit (Qiagen) with DNAse I treatment (Qiagen) according to manufacturer’s instructions. RNA was quantified and quality assessed using Agilent 2100 bioanalyser.

### Microarray Analysis

RNA was amplified, converted to double stranded cDNA and biotin labelled according to Affymetrix protocols (Affymetrix, Ca, USA). Biotinylated cDNA was hybridised to Human U133 plus 2.0 chips (Affymetrix). The gene chips were scanned using an Agilent gene array scanner 3000 and fluorescence intensity was analysed using gene chip operating software (Affymetrix). Quality control of the gene chips was checked using AffyQCreport R software (Bioconductor.org). The full datasets have been submitted to arrayexpress (Accession number E-MTAB-1688).

### PCR

50 ng of RNA was reverse transcribed using the Verso 1-step QRT-PCR mix kit (Thermo-Scientific, Surrey, UK) according to manufacturer’s instructions. Genomic DNA contamination was monitored with no-RNA control reactions. TaqMan Reverse transcription PCR was performed in duplicate for each sample using 1 µl of cDNA in a 25 µl reaction in step 2 of the Verso 2-step QRT-PCR mix kit (Thermo-scientific) containing 0.5 µl of premade ABI TaqMac gene expression assays for CD247 (Hs00609525_m1, Applied Biosystems, Warrington, UK), Linker of activated T cells (LAT, Hs01065378_g1, Applied Biosystems), LCK (Hs00178427_m1, Applied Biosystems), VAV2 guanine nucleotide exchange factor (VAV2, Hs00610104_m1, Applied Biosystems) and VAV3 guanine nucleotide exchange factor (VAV3, Hs00196125_m1, Applied Biosystems) and the endogenous control was 18s ribosomal RNA (18s, Hs99999901_s1, Applied Biosystems). Gene expression levels were calculated using the comparative CT method normalised to 18s.

### Immunofluorescence

Cells were fixed in frozen methanol (Sigma-Aldrich), washed in phosphate buffered saline and then blocked with 1.5% goat serum (Vector Laboratories, Peterborough, UK). Cells were then incubated overnight at 4°C in a cocktail of mouse anti-CD8 (1 in 200, C8/144B, Dako, Cambridgeshire, UK) and rabbit anti-CD247 (1 in 1000, HPA008750, Sigma-Aldrich) diluted in goat serum. Cells were washed and then incubated with a cocktail of alexa-488 conjugated goat anti-mouse secondary antibody (Invitrogen, Paisley, UK) and alexa-568 conjugated goat anti-rabbit secondary antibody (Invitrogen) for 90 minutes at 37°C. Cells were washed, nuclei counterstained with 4′,6-diamidino-2-phenylindole (DAPI) and mounted with vectashield (Vector laboratories). CD8 positive cells and dual-labelled CD8+, CD247+ cells were counted using a Nikon eclipse 80i microscope (Nikon UK ltd, Surrey, UK with QImaging digital camera and Image Proplus software (Media cybernetics, Marlow, UK). Alexa-488 (green) and Alexa-568 (red) conjugated secondary antibodies were visualised using exciter filters with band widths of 465–495 nm and 540–580 nm respectively. For dual-label images, fluorescent images from the same field were captured and digitally merged to identify CD8 staining cells (green) that co-stained with CD247 (red). Images were obtained of all identified CD8 cells. Cell counting was performed by an individual blinded to the patient information until after cell counting was complete.

### Statistics

Differential gene expression analysis was performed by linear modelling using the lmFit and eBayes functions of the Limma software package [20]. Comparisons were made between COPD pulmonary and peripheral blood samples and also between COPD and S pulmonary samples. False discovery rate errors were controlled for using the Q value method [21]; A Q value of 0.01 was assigned as statistically significant.

Principal component analysis (PCA) was performed to compare COPD pulmonary samples with paired blood samples and also COPD pulmonary samples with S pulmonary samples. Arrays were plotted on a 2-dimensional image of principal components to show trends in gene expression.

The list of genes differentially expressed between paired blood and pulmonary COPD samples (fold change>2, Q<0.01) was inputted into the Database for Annotation, Visualisation and Integrated Discovery (DAVID) [22]. This database links genes with known annotation terms from numerous public databases including GO terms, Kyoto Encyclopaedia for Genes and Genomes (KEGG) and Biocarta. The functional annotation clustering tool was used to define groups of related annotation terms enriched within the input gene list. Clusters were ranked according to the enrichment score which is based on the modified fishers p value when compared to background expression.

The genes identified within the cluster of interest were input into the Search Tool for Retrieval of Interacting Genes/proteins (STRING). This database creates networks of genes based upon known or predicted protein-protein interactions [23]. This allows clear visualisation of the ways in which genes interact with each other making it possible to better understand the biological relationships between genes.

Due to the small group sizes it was not possible to accurately test all datasets for normality. Therefore data was analysed using non-parametric tests. Gene expression levels measured by PCR were compared using Mann-Whitney U test. Immunoflourescence data were compared between groups using Kruskal-Wallis test, with subsequent pairwise comparisons using Dunn’s multiple comparisons test if Kruskal-Wallis p<0.05. For PCR and immunofluorescence data, p<0.05 was considered statistically significant. Statistical analysis was performed using Graphpad inStat (Graphpad Sofware inc. Ca, USA).

## Results

### Gene Expression Analysis

The procedure to isolate CD8 cells from COPD and S lung tissue resulted in a near-pure population, with 90.2% and 89.8% (mean values) CD8 cells positive for CD8 by immunofluorescence. The isolation of CD8 cells from COPD blood samples resulted in marginally higher CD8 purity (mean 95.1%).

### Principal Component Analysis

**Figure 1:**
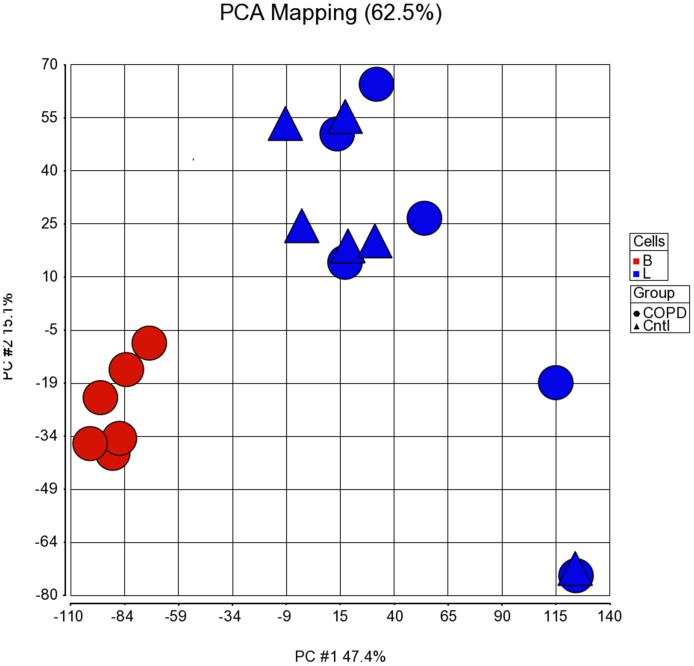
Principal component analysis of COPD blood and pulmonary CD8 cells and smoker pulmonary CD8 cells. Principal component analysis of the micro-array data sets for paired blood (red circles) and pulmonary (blue circles) COPD CD8 cells (n = 6) and pulmonary smoker CD8 cells (Blue triangles, n = 6) was performed. Principal components 1 and 2 are charted accounting for 62.5% of all variation.

PCA showed a clear separation between clusters relating to COPD pulmonary and peripheral blood CD8 samples. PCA showed no significant variation between COPD and S pulmonary samples (Figure 1).

### Differentially Expressed Genes in COPD Pulmonary and Peripheral Blood Samples

There were 2938 probe sets accounting for 1924 genes that were differentially expressed between paired COPD pulmonary and peripheral blood samples, and the most highly regulated genes are shown in table S1. The majority of these genes were not specific for lymphocyte activation and signalling. These findings are possibly explained by the presence of contaminating cells in the pulmonary samples.

**Figure 2:**
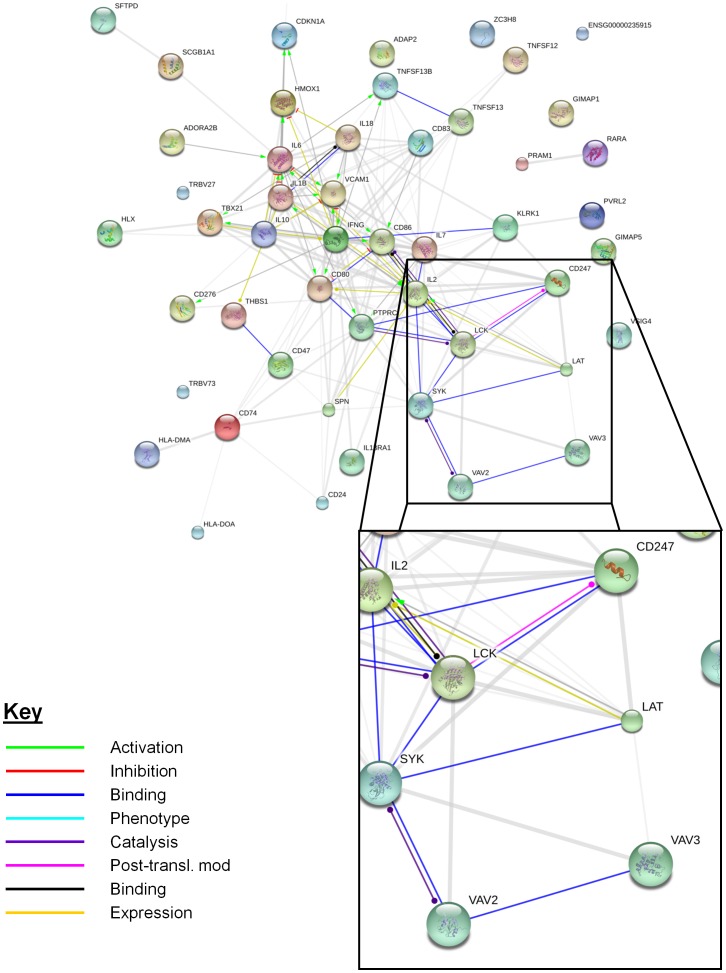
STRING network representing interactions between genes within lymphocyte specific functional annotation cluster. The 51 genes within the functional annotation cluster were input into the STRING database. A network of how each gene interacts with others was produced. A sub-group of genes which interact closely, primarily through binding to each other, was highlighted (inset image). The genes of interest were CD247; LCK: Lymphocyte-specific protein tyrosine kinase; LAT: Linker of activated T cells; SYK: spleen tyrosine kinase; VAV2: VAV2 guanine nucleotide exchange factor; VAV3: VAV3 guanine nucleotide exchange factor.

DAVID was used to identify clusters of genes specifically associated with T cell activation or signalling; one cluster was identified that was composed of genes specific to T cell function. There were a total of 51 genes within the entire cluster (Table 2) comprising 8 related gene ontology terms (table S2). A number of genes within the entire cluster related to more than one gene ontology term. The gene list was input into the STRING database in order to better understand the biological relationships between the genes within this cluster (Figure 2). The network revealed a sub-group of genes within this cluster that interact closely with each other (highlighted in figure 2). These genes are components of the TCR signalling pathway. The expression levels of CD247 (fold change −2.43, Q<0.001), LCK (fold change −2.25, Q<0.01), LAT (fold change −2.22, Q = 0.01), protein tyrosine phosphatise receptor C (PTPRC, fold change −2.33, Q = 0.002) and VAV3 (fold change −2.87, Q<0.001) were decreased in pulmonary samples compared to peripheral blood samples. VAV2 (fold change +2.87, Q<0.001), and spleen tyrosine kinase (Syk, fold change +5.28, Q<0.001) were expressed at higher levels in pulmonary samples.

**Table 2:** Genes identified within functional annotation cluster relating to T Cell Receptor signalling.

Affymetrix ID	Gene name	Foldchange	Q value
205725_at	secretoglobin, family 1A, member 1 (uteroglobin)	381.82	<0.0001
204787_at	V-set and immunoglobulin domain containing 4	92.12	<0.0001
210511_s_at, 227140_at	inhibin, beta A	52.48	<0.0001
203381_s_at, 203382_s_at, 212884_x_at	hypothetical LOC100129500; apolipoprotein E	34.31	0.0002
211612_s_at, 210904_s_at, 201887_at, 201888_s_at	interleukin 13 receptor, alpha 1	23.95	<0.0001
210895_s_at, 205686_s_at, 205685_at	CD86 molecule	18.63	0.0001
201110_s_at, 201108_s_at, 201109_s_at, 235086_at	thrombospondin 1	15.99	0.0002
232068_s_at, 1552798_a_at, 224341_x_at	toll-like receptor 4	15.55	0.0004
203665_at	heme oxygenase (decycling) 1	15.22	0.0002
205067_at, 39402_at	interleukin 1, beta	15.01	0.0001
214199_at	surfactant protein D	12.56	0.0002
205207_at	interleukin 6 (interferon, beta 2)	11.27	0.0002
209499_x_at, 211495_x_at, 209500_x_at, 210314_x_at	TNFSF12-TNFSF13 readthrough transcript; tumor necrosis factor (ligand)superfamily, member 12; tumor necrosis factor (ligand) superfamily,member 13	11.13	0.002
204440_at	CD83 molecule	10.88	<0.0001
206295_at	interleukin 18 (interferon-gamma-inducing factor)	10.56	0.0003
210354_at	interferon, gamma	7.32	0.0004
216379_x_at	CD24 molecule; CD24 molecule-like 4	7.12	0.003
202284_s_at	cyclin-dependent kinase inhibitor 1A (p21, Cip1)	6.01	<0.0001
217478_s_at	major histocompatibility complex, class II, DM alpha	5.56	0.0006
207849_at	interleukin 2	5.36	0.0008
241742_at	PML-RARA regulated adaptor molecule 1	5.27	0.0007
209619_at, 1567628_at, 241849_at	CD74 molecule, major histocompatibility complex, class II invariant chain	4.82	0.0003
215633_x_at 214574_x_at, 211581_x_at, 210629_x_at, 211582_x_at, 214181_x_at	leukocyte specific transcript 1	4.67	0.002
226068_at, 207540_s_at	spleen tyrosine kinase	4.55	0.003
1554519_at, 1555689_at, 207176_s_at	CD80 molecule	4.40	0.002
223501_at, 223502_s_at	tumor necrosis factor (ligand) superfamily, member 13b	4.32	0.0007
205891_at	hypothetical LOC100131909; adenosine A2b receptor	4.02	0.0009
203868_s_at	vascular cell adhesion molecule 1	3.95	0.008
226878_at	major histocompatibility complex, class II, DO alpha	3.87	0.007
228037_at, 203749_s_at	retinoic acid receptor, alpha	3.64	0.0008
241808_at	interleukin 7	3.44	0.0008
224859_at	CD276 molecule	3.23	0.0004
207433_at	interleukin 10	2.92	0.001
214438_at	H2.0-like homeobox	2.73	0.0005
203140_at, 228758_at, 236439_at	B-cell CLL/lymphoma 6	2.66	0.0002
203149_at	poliovirus receptor-related 2 (herpesvirus entry mediator B)	2.00	0.004
1568964_x_at	Sialophorin	−2.03	0.02
220684_at	T-box 21	−2.22	0.04
204890_s_at	lymphocyte-specific protein tyrosine kinase	−2.22	0.01
211005_at	linker for activation of T cells///spinster homolog 1 (Drosophila)	−2.25	0.01
239467_at	protein tyrosine phosphatase, receptor type, C	−2.34	0.002
242974_at	CD47 molecule	−2.35	0.001
211902_x_at, 234849_at	T cell receptor alpha locus	−2.36	0.009
210031_at	CD247 molecule	−2.43	0.001
234377_at	T cell receptor beta variable 7–8	−2.50	0.006
1555691_a_at	killer cell lectin-like receptor subfamily K, member 1	−2.72	0.004
239644_at	zinc finger CCCH-type containing 8	−2.73	0.0006
218805_at, 64064_at	GTPase, IMAP family member 5	−3.43	0.0002
1552318_at, 1552316_a_at, 1552315_at, 236401_at	GTPase, IMAP family member 1	−3.55	<0.0001

Affymetrix ID: Affymetrix GeneChip Human Genome U133 plus 2.0 probe set identifier; Fold Change: Fold change in gene expression from peripheral blood to pulmonary samples.

Natural killer (NK) cells can co-express CD8 but do not express TCR. NK cell markers (CD56, CD16, NCR1, NCR2, NCR3 and NKG2D) were either similar between blood and lung or significantly higher in the blood samples, ruling out the possibility of reduced TCR gene expression in pulmonary CD8 cells due to a higher proportion of NK cells in the pulmonary preparations (Table S3).

### Differentially Expressed Genes in COPD and Smokers Lung Samples

There were no statistically significant differences between the gene expression profile of the isolated cells from COPD and S pulmonary samples in the microarray analysis. However, the expression levels of the TCR genes which were down-regulated in pulmonary compared to blood samples revealed fold changes showing decreased expression of CD247 (fold change −1.4, Q = 0.81) and LCK (fold change −1.5, Q = 0.77) in COPD pulmonary CD8 cells compared to S pulmonary CD8 cells, but Q values that were non-significant.

### PCR Analysis

**Figure 3:**
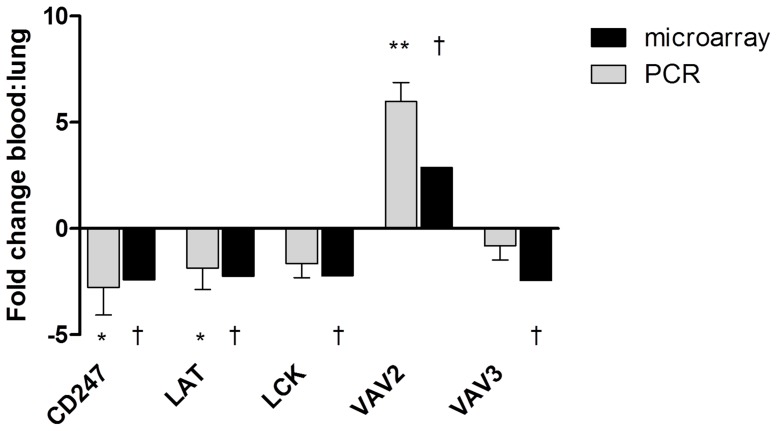
Comparison of blood to lung fold change of T cell receptor signalling genes measured by micro-array and PCR. Gene expression levels of CD247, Leukocyte-specific protein tyrosine kinase (LCK); Linker of activated T cells (LAT); VAV2 guanine nucleotide exchange factor (VAV2) and VAV3 guanine nucleotide exchange factor (VAV3) were measured by both human U133 plus 2 affymetrix gene assay and by quantitative reverse transcription polymerase chain reaction (PCR). Fold change from blood to lung is charted. Statistically significant differences between blood and pulmonary levels measured by PCR are indicated by *p<0.05, **p<0.001. Statistically significant differences between blood and pulmonary levels measured by microarray are indicated by † Q<0.01.

Quantitative real time PCR was performed for CD247, LCK, LAT, VAV2 and VAV3; these 5 genes relate specifically to TCR signalling and were differentially regulated between COPD pulmonary and blood CD8 cells in the microarray analysis. PCR gene expression levels were significantly different for CD247 (fold change −2.77, p = 0.001), LAT (fold change −1.86, p = 0.01) and VAV2 (fold change 5.99, p<0.001) between COPD pulmonary and blood cells (Figure 3). The difference for LCK approached statistical significance (fold change −1.66, p = 0.06), but there was no difference for VAV3 (p = 0.16).

**Figure 4:**
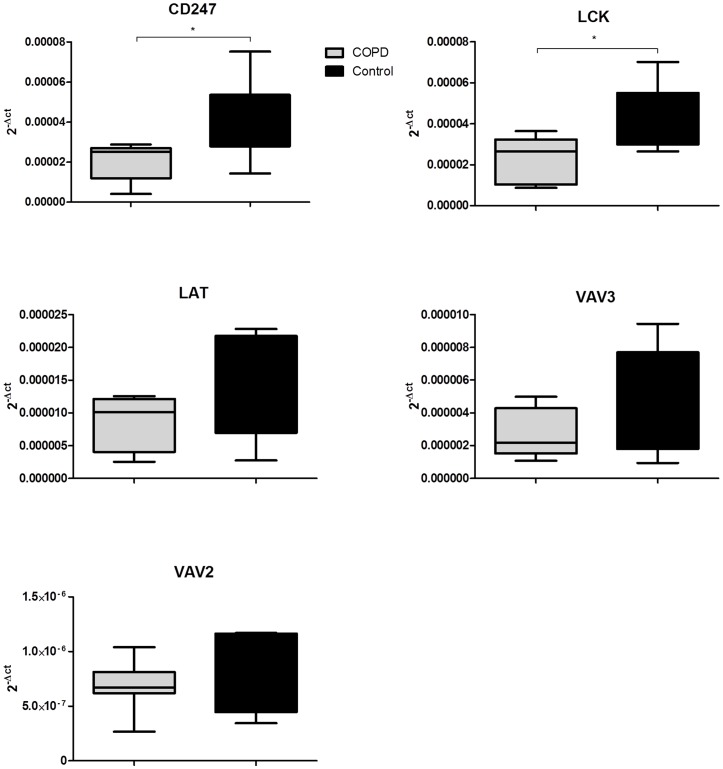
Transcript levels of TCR signalling genes in COPD and Smoker pulmonary CD8 cells. Pulmonary CD8 cells were isolated from chronic obstructive pulmonary disease (COPD, grey n = 6) and smokers with normal lung function (S, black, n = 6). Transcript levels of CD247, Leukocyte-specific protein tyrosine kinase (LCK); Linker of activated T cells (LAT); VAV2 guanine nucleotide exchange factor (VAV2) and VAV3 guanine nucleotide exchange factor (VAV3) were measured by quantitative real time polymerase chain reaction. Statistically significant differences between COPD and S are represented by *p<0.05.

Although analysis of the microarray dataset did not reveal any statistically significant differences between COPD and S pulmonary samples, this may be related to the relatively high false negative rate associated with multiple testing in micro-array analysis. Therefore, we analysed the 5 gene panel (CD247, LCK, LAT, VAV2 and VAV3) by applying PCR to the COPD and S pulmonary samples; CD247 (fold change −1.79, relative reduction 46% p = 0.006) and LCK (fold change −1.77, relative reduction 44% p = 0.03) were both expressed at significantly lower levels in COPD compared to S pulmonary CD8 cells (Figure 4). There were no significant differences for gene expression levels of LAT, VAV2 or VAV3.

### Immunofluorescence of CD247 in Pulmonary CD8 Cells

CD247 was chosen for protein analysis in a separate cohort of patients as this protein was significantly differentially expressed between blood and lung COPD CD8 cells and also down-regulated in COPD compared to S pulmonary CD8 cells. Furthermore, CD247 is known to be down-regulated in other autoimmune diseases [16].

**Figure 5:**
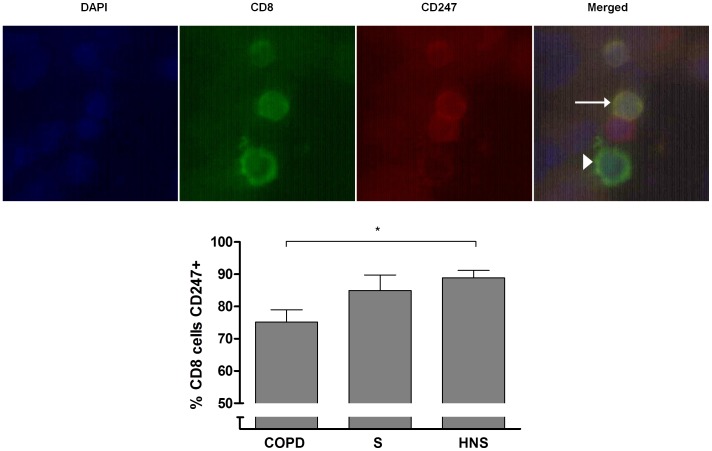
The expression of CD247 by airway CD8 cells. Bronchoalveolar lavage (BAL) was obtained from chronic obstructive pulmonary disease (COPD, n = 7), smokers with normal lung function (S, n = 4) and healthy non-smokers (HNS, n = 8). Cells were labelled with anti-CD8 (green) and anti-CD247 (red). Nuclei were counterstained with 4′,6-diamidino-2-phenylindole (DAPI, blue). Top panel: Representative images of COPD BAL cells. Merged image displays a CD8+CD247+ cell (yellow, arrow), a CD8+CD247- cell (green, arrow head) and a CD8-CD247+ (red). Lower panel: Percentage of BAL CD8 cells positive for CD247 in COPD, S and HNS. Statistical significance indicated by *p<0.05. Data was analysed by Kruskall-Wallis test followed by Dunn’s multiple comparisons test if p<0.05.

BAL cytospins from 7 COPD patients, 5 S and 8 HNS were dual stained for CD8 and CD247 (figure 5). A median (range) of 32.5 (15–216) CD8 cells were analysed for CD247 expression. A lower proportion of CD8 cells from COPD patients were positively immunostained for CD247 compared to S and HNS; median 73.1, 83.8 and 89.1% respectively (Kruskal-Wallis p = 0.02, Dunn’s multiple comparisons test COPD vs HNS p<0.05). CD247 expression was noted on cells that did not stain for CD8; these were most likely to be CD4 cells as CD247 is known to be expressed on this cell type [24].

## Discussion

Our understanding of the phenotype of CD8 cells within COPD lung remains limited. We now show that components of the TCR in COPD pulmonary CD8 cells, including CD247, LCK, LAT and VAV3, have reduced expression compared to circulating CD8 cells in the same individuals. These changes in CD8 gene expression may be due to local factors unique to the lung microenvironment. We also demonstrate down regulation of CD247 and LCK in COPD compared to S pulmonary CD8 cells using qRT-PCR and immunofluorescence. It appears that pulmonary CD8 cells from COPD patients have down-regulated expression of key components of the TCR signalling pathway compared to controls.

CD247 (or TCR-ζ) is a transmembrane protein with a number of immunoreceptor tyrosine-based activation motifs (ITAMs) that plays a key role in both transduction of TCR signalling and also in anchoring the TCR to the cell membrane [25]. Down regulation of CD247 and LCK has been associated with other autoimmune diseases, tumours and chronic infections all of which are associated with persistent exposure to antigen [18]. This process is, at least in part, driven by the function of the regulatory myeloid suppressor cell [26]. These cells are thought to decrease expression of CD247 by metabolising l-arginine which is required for CD247 expression. Increased numbers of myeloid suppressor cells within COPD lungs may therefore decrease CD247 expression [27]. The transient down-regulation of CD247 occurs after time-limited exposure to antigen, such as during an acute viral infection [28]. This is thought to be a physiological regulatory mechanism of the adaptive immune system to allow it to de-escalate following acute activation. However, with persistent exposure to antigen, such as in auto-immune diseases, CD247 is unable to return to normal levels. This leads to an immunosuppressive state associated with T cell dysfunction [16]. The down regulation of CD247 does not just occur in T cells specific to the offending antigen; there is a bystander effect with down regulation of CD247 in all T cells [29].

The onset of COPD is associated with an increase in CD8 numbers [8], [9], and it can be hypothesized that CD8 cells contribute to the pathogenesis of COPD through their cytotoxic effect [13], [30] and by release of pro-inflammatory cytokines [12], [31]. However, we have demonstrated decreased expression of molecules associated with TCR, suggesting decreased TCR associated effector function in COPD CD8 cells. The TCR is responsible for antigen specific responses, while it is known that CD8 cells can be activated through non-antigen specific mechanisms such as cytokine stimulation [32] or TLR signalling [33]. TLR expression is increased on COPD pulmonary CD8 cells [34], [35], and these cells release more cytokine than control pulmonary CD8 cells when co-stimulated with a TLR1/2 ligand [35]. This non-antigen specific activation of CD8 cells could account for some of the increased effector functions seen in COPD. In contrast, the down regulation of CD247 suggests an inadequate ability to respond specifically to pathogens, and may account for the predisposition to recurrent airway infections in patients with COPD [36].

There were no statistically significant differences found between COPD and smoker control pulmonary CD8s in the microarray data. The nature of the statistical analysis of large microarray datasets means that correction for multiple testing must be made. In a limited sample size, it is inevitable that false negative findings will occur using this approach. The PCR and immunohistochemistry analysis performed did not require such stringent correction for multiple testing, and so were able to demonstrate significantly lower levels of CD247 and LCK in COPD compared to S pulmonary CD8 cells.

LCK is a member of the Src family of tyrosine kinases which controls the initiation and amplification of TCR signalling. Antigen binding to the TCR promotes LCK recruitment to the TCR complex where it phosphorylates ITAMs in the CD247 complex thus beginning the TCR signalling cascade [37]. LCK has been shown to mediate some of the immediate non-genomic immunosuppressive effects of glucocorticoids [38], [39]. Diminished expression of LCK could thus relate to the limited effect of glucocorticoids that we have previously reported in COPD pulmonary T cells [40], although this has not been studied.

Natural killer cells can co-express CD8 but do not express the T cell receptor. Increased numbers of NK cells within the pulmonary samples compared to the peripheral blood samples could be an explanation for the key findings within the current studies. However, there were no significant differences in the microarray data of levels of NK cell markers such as CD56 suggesting no difference in NK cell levels between the samples.

The results reported here concern the expression of CD8 genes, with validation of CD247 by protein analysis. We have not studied the functional consequences of the observed changes in gene expression. It will be important to understand whether the reduction in the expression of TCR associated components in COPD CD8 cells are associated with a loss of function. This may be important clinically; for example, when considering the ability of CD8 cells to mount an antigen specific response to pathogens.

We used immunofluorescence to evaluate CD247 expression using samples that had been historically collected for research purposes. An alternative method to evaluate CD247 in bronchoscopy samples is flow cytometry, which is potentially more sensitive as more cells are counted. However, flow cytometry requires fresh samples to analyse immediately after collection, which was not possible with our historic samples. Nevertheless, the immunofluorescence analysis for CD247 provided results compatible with the reduction in expression observed by PCR in COPD patients.

The gene expression study using lung surgical tissue had a limited sample size, but was sufficiently large enough to identify candidate genes by microarray that were validated using PCR and immunohistochemistry. The limited sample size meant that the effect of clinical variables such as smoking pack year history and severity of disease could not be accounted for. Furthermore, the practicalities of obtaining lung tissue from patients undergoing cancer surgery preclude the collection of accurate COPD phenotype data such as emphysema assessment by high resolution CT scan, as the patients are usually “fast tracked” to surgery leaving insufficient time to perform extensive research procedures. Furthermore, the cancer itself can impact on COPD symptoms such as exacerbation rates or breathlessness. Nevertheless, the relationship between COPD clinical characteristics and CD8 phenotype is of interest, but should be addressed in studies that obtain cells from COPD patients without lung cancer.

It would have been preferable to include a group of never smokers in the gene expression analysis, but no such patients underwent surgery for lung cancer during the course of the study. Only a small number of never smokers develop lung cancer, which made it practically very difficult to include such a group. We used historically collected samples from bronchoscopy to evaluate CD247 protein, and were able to include a group of non-smokers in this analysis.

In the gene expression study, it should be noted that all the COPD patients and controls were ex-smokers; this ensured that there was no confounding effect of current cigarette smoking on gene expression. In the bronchoscopy study, the COPD patients were all current smokers, and it is possible that current smoking may have an effect on CD8 gene expression. Nevertheless, there was a difference in CD247 expression in COPD patients compared to HNS, while this was not observed between S and HNS; this suggests the downregulation of CD247 occurs because of COPD and not current cigarette smoking.

It has been assumed that an increase in the pro-inflammatory and cytotoxic activity of CD8 cells is the principal mechanism by which these cells contribute to the pathophysiology of COPD [4], [5]. There is increasing evidence that non-antigen specific stimulation is responsible for the activation of these cells [34], [35]. We now show that key T cell receptor signalling molecules are down regulated in pulmonary CD8 cells, leading to the hypothesis that dysfunction of the antigen specific response of CD8 cells in COPD predisposes to recurrent infections. This possibility should be further investigated, and may represent a potential therapeutic avenue for novel therapeutic interventions designed to prevent COPD exacerbations.

## Supporting Information

Table S1 **Most significantly regulated genes between pulmonary and peripheral blood samples.**
(DOCX)

Table S2 **Annotation terms within Functional Annotation Cluster.**
(DOCX)

Table S3 **Expression of natural killer cell markers in blood and lung samples.**
(DOCX)

Methods S1 **Detailed methods for the isolation of pulmonary lymphocytes from explanted human lung.**
(DOCX)
