# Supplementary material for: Down Regulation of T Cell Receptor Expression in COPD Pulmonary CD8 Cells
Source: PLoS One. 2013 Aug 19;8(8):e71629. doi: 10.1371/journal.pone.0071629 (PMC3747211; doi:10.1371/journal.pone.0071629)
Supplement: Table S1 — Most significantly regulated genes between pulmonary and peripheral blood samples. (DOCX) [file pone.0071629.s001.docx]

|  | **Gene Title** | **Fold Change** | **Cellular Source** |
| --- | --- | --- | --- |
| GPNMB | glycoprotein (transmembrane) nmb | 685.4 |  |
| FN1 | fibronectin 1 | 636.7 |  |
| CCL18 | chemokine (C-C motif) ligand 18 (pulmonary and activation-regulated) | 505.5 | macrophage |
| FABP4 | fatty acid binding protein 4, adipocyte | 496.7 |  |
| MRC1 /// MRC1L1 | mannose receptor, C type 1 /// mannose receptor, C type 1-like 1 | 442.3 | macrophage |
| SCGB1A1 | secretoglobin, family 1A, member 1 (uteroglobin) | 381.8 |  |
| OLR1 | oxidized low density lipoprotein (lectin-like) receptor 1 | 320.8 | endothelium |
| MS4A4A | membrane-spanning 4-domains, subfamily A, member 4 | 193.6 |  |
| C1QC | complement component 1, q subcomponent, C chain | 170.5 |  |
| IFI27 | interferon, alpha-inducible protein 27 | 145.1 |  |
| PCOLCE2 | procollagen C-endopeptidase enhancer 2 | 138.6 |  |
| LPL | lipoprotein lipase | 131.1 |  |
| MSR1 | macrophage scavenger receptor 1 | 123.0 | macrophage |
| CXCL3 | chemokine (C-X-C motif) ligand 3 | 113.7 | macrophage |
| SERPING1 | serpin peptidase inhibitor, clade G (C1 inhibitor), member 1 | 106.1 |  |
| SFTPA2 | surfactant protein A2 | 105.3 | Alveolar cell |
| B3GNT5 | UDP-GlcNAc:betaGal beta-1,3-N-acetylglucosaminyltransferase 5 | 102.4 |  |
| ITGB8 | integrin, beta 8 | 100.4 |  |
| GPNMB | glycoprotein (transmembrane) nmb | 97.2 |  |
| VSIG4 | V-set and immunoglobulin domain containing 4 | 92.1 |  |
| CXCL2 | chemokine (C-X-C motif) ligand 2 | 90.9 | Monocytes, neutrophils |
| BHLHE41 | basic helix-loop-helix family, member e41 | 90.6 |  |
| CCL18 | chemokine (C-C motif) ligand 18 (pulmonary and activation-regulated) | 88.7 | DC/macrophage |
| MARCO | macrophage receptor with collagenous structure | 87.5 | Macrophages |
| SCGB3A2 | secretoglobin, family 3A, member 2 | 85.2 |  |

Cellular source: Cell types known to be the predominant cell to express the gene; Fold change: Fold change in gene expression from blood to pulmonary samples.
