# Supplementary material for: Down Regulation of T Cell Receptor Expression in COPD Pulmonary CD8 Cells
Source: PLoS One. 2013 Aug 19;8(8):e71629. doi: 10.1371/journal.pone.0071629 (PMC3747211; doi:10.1371/journal.pone.0071629)
Supplement: Table S2 — Annotation terms within Functional Annotation Cluster. (DOCX) [file pone.0071629.s002.docx]

| **Annotation Term** | **Gene Count** | **P value** |
| --- | --- | --- |
| [positive regulation of cell activation](http://www.ebi.ac.uk/QuickGO/GTerm?id=GO:0050867) | 33 | 2.4E-7 |
| [positive regulation of leukocyte activation](http://www.ebi.ac.uk/QuickGO/GTerm?id=GO:0002696) | 32 | 2.5E-7 |
| [regulation of cell activation](http://www.ebi.ac.uk/QuickGO/GTerm?id=GO:0050865) | 44 | 3.4E-7 |
| [regulation of leukocyte activation](http://www.ebi.ac.uk/QuickGO/GTerm?id=GO:0002694) | 42 | 5.4E-7 |
| [positive regulation of lymphocyte activation](http://www.ebi.ac.uk/QuickGO/GTerm?id=GO:0051251) | 29 | 1.3E-6 |
| [regulation of lymphocyte activation](http://www.ebi.ac.uk/QuickGO/GTerm?id=GO:0051249) | 37 | 3.8E-6 |
| [positive regulation of T cell activation](http://www.ebi.ac.uk/QuickGO/GTerm?id=GO:0050870) | 24 | 4.6E-6 |
| [regulation of T cell activation](http://www.ebi.ac.uk/QuickGO/GTerm?id=GO:0050863) | 31 | 7.9E-6 |

Gene count: number of genes within list of differentially expressed genes associated with annotation term; P value calculated by comparison between gene list and background expression using the Benjamani-Hochberg method for multiple comparisons.
