# Supplementary material for: Down Regulation of T Cell Receptor Expression in COPD Pulmonary CD8 Cells
Source: PLoS One. 2013 Aug 19;8(8):e71629. doi: 10.1371/journal.pone.0071629 (PMC3747211; doi:10.1371/journal.pone.0071629)
Supplement: Table S3 — Expression of natural killer cell markers in blood and lung samples. (DOCX) [file pone.0071629.s003.docx]

| **Gene Symbol** | **Fold Change (blood to lung)** | **Q value** |
| --- | --- | --- |
| CD56 | -2.15 | 0.042 |
| CD16 | 1.015907 | 0.52 |
| NCR1 | -2.64843 | 0.0016 |
| NCR2 | -1.05728 | 0.27 |
| NCR3 | -2.85934 | 0.0040 |
| NKG2D | -2.71834 | 0.0040 |

CD-: cluster of differentiation ; NCR: natural cytotoxicity triggereing receptor; NKG2D: Natural Killer cell receptor D.
