# Supplementary material for: Down Regulation of T Cell Receptor Expression in COPD Pulmonary CD8 Cells
Source: PLoS One. 2013 Aug 19;8(8):e71629. doi: 10.1371/journal.pone.0071629 (PMC3747211; doi:10.1371/journal.pone.0071629)
Supplement: Methods S1 — Detailed methods for the isolation of pulmonary lymphocytes from explanted human lung. (DOCX) [file pone.0071629.s004.docx]

**Supplementary Methods**

**Isolation of pulmonary lymphocytes**

Explanted lung was homogenised in a BL650 blender (Kenwood, Hampshire, UK) with 50ml of RPMI-1640 (Sigma-Aldrich, Dorset, UK) for 45 seconds at 13000 rpm. Cells were then passed through a 100µm filter (Millipore, Watford, UK) and the resultant cell suspension was centrifuged to obtain a cell pellet. The cell pellet was resuspended in isotonic 40% Percoll (GE Healthcare). The cell suspension was layered over isotonic 70% Percoll and centrifuged for 30 minutes at 800g, 4^°^C. The top layer consisting predominantly of macrophages and dead cells was removed and the interface layer of enriched lymphocytes was taken and resuspended in 50mls RPMI-1640 (Sigma-Aldrich) prior to centrifugation (10 minutes, 400g, 4^°^C) to obtain a cell pellet. The cell pellet was resuspended in Phosphate-buffered saline (Sigma-Aldrich) containing 2mM ethylenediaminetetraacetic acid (EDTA) and 0.5% Fetal Calf Serum (FCS) prior to isolation of CD8 cells using CD8 microbeads according to manufacturers instructions (Miltenyi biotec).
